# The Impact of Ambivalent Attitudes on the Helpfulness of Web-Based Reviews: Secondary Analysis of Data From a Large Physician Review Website

**DOI:** 10.2196/38306

**Published:** 2023-05-29

**Authors:** Wei Dong, Yongmei Liu, Zhangxiang Zhu, Xianye Cao

**Affiliations:** 1 Business School Central South University Changsha China; 2 Department of Information Systems City University of Hong Kong Hong Kong Hong Kong; 3 College of Tourism Hunan Normal University Changsha China; 4 School of Business Administration Hunan University of Technology and Business Changsha China

**Keywords:** web-based review helpfulness, ambivalent attitudes, risk reduction, the tripartite model of attitudes, mobile phone

## Abstract

**Background:**

Previously, most studies used 5-star and 1-star ratings to represent reviewers’ positive and negative attitudes, respectively. However, this premise is not always true because individuals’ attitudes have more than one dimension. In particular, given the credence traits of medical service, to build durable physician-patient relationships, patients may rate their physicians with high scores to avoid lowering their physicians’ web-based ratings and help build their physicians’ web-based reputations. Some patients may express complaints only in review texts, resulting in ambivalence, such as conflicting feelings, beliefs, and reactions toward physicians. Thus, web-based rating platforms for medical services may face more ambivalence than platforms for search or experience goods.

**Objective:**

On the basis of the tripartite model of attitudes and uncertainty reduction theory, this study aims to consider both the numerical rating and sentiment of each web-based review to explore whether there is ambivalence and how ambivalent attitudes influence the helpfulness of web-based reviews.

**Methods:**

This study collected 114,378 reviews of 3906 physicians on a large physician review website. Then, based on existing literature, we operationalized numerical ratings as the cognitive dimension of attitudes and sentiment in review texts as the affective dimension of attitudes. Several econometric models, including the ordinary least squares model, logistic regression model, and Tobit model, were used to test our research model.

**Results:**

First, this study confirmed the existence of ambivalence in each web-based review. Then, by measuring ambivalence through the inconsistency between the numerical rating and sentiment for each review, this study found that the ambivalence in different web-based reviews has a different impact on the helpfulness of the reviews. Specifically, for reviews with positive emotional valence, the higher the degree of inconsistency between the numerical rating and sentiment, the greater the helpfulness is (β_positive 1_=.046*; P*<.001). For reviews with negative and neutral emotional valence, the impact is opposite, that is, the higher the degree of inconsistency between the numerical rating and sentiment, the lesser the helpfulness is (β_negative 1_=−.059, *P*<.001; β_neutral 1_=−.030, *P*=.22). Considering the traits of the data, the results were also verified using the logistic regression model (θ_positive 1_=0.056, *P*=.005; θ_negative 1_=−0.080, *P*<.001; θ_neutral 1_=−0.060, *P*=.03) and Tobit model.

**Conclusions:**

This study confirmed the existence of ambivalence between the cognitive and affective dimensions in single reviews and found that for reviews with positive emotional valence, the ambivalent attitudes lead to more helpfulness, but for reviews with negative and neutral emotion valence, the ambivalence attitudes lead to less helpfulness. The results contribute to the web-based review literature and inspire a better design for rating mechanisms in review websites to enhance the helpfulness of reviews.

## Introduction

### Background

With the development of eHealth, increasingly more patients share their clinical experiences or use web-based reviews to evaluate physicians before making their choices [1,2]. A survey by Hedges and Couey [3] stated that 90% of patients use web-based reviews to evaluate their physicians, and 71% of these patients first refer to web-based reviews when they seek a new physician. Dunivin et al [4] found that web-based reviews can inform patients’ choice of physicians and thus affect both patients and physicians. Lu and Wu [5] found that the overall rating and number of reviews can influence physicians’ outpatient visits. Grabner-Kräuter and Waiguny [6] conducted an experiment and found that web-based reviews can influence patients’ attitudes toward the rated physicians. Lin et al [7] collected web-based review data from Healthgrades and found that web-based patient reviews could be used as a data source for understanding patient experiences and the health care quality in dentistry. Gao et al [8] also confirmed that physicians’ web-based ratings are positively related to how offline patients perceive their quality. Thus, web-based ratings and physician quality are positively correlated [9]. Because physicians’ web-based reviews have a substantial impact on patients, physicians’ web-based reviews have an indirect impact on physicians. For example, a 2015 survey found that 53% of physicians had visited physicians’ review websites at least once, and 78% of physicians believed that web-based reviews increased their job stress [10]. Emmert et al [11] also investigated an increasing trend in which physicians respond to patients’ web-based ratings.

However, although web-based reviews are important for both patients and physicians, they have several drawbacks. Only 11% of patients rate their physicians negatively, and most reviews are either positive or neutral [1,3]. This phenomenon is consistent with the J shape of web-based ratings, indicating that web-based ratings concentrate on high scores (eg, 5 stars) [12]. The distribution of web-based ratings is not helpful for later patients to distinguish excellent physicians from ordinary physicians, resulting in inefficient web-based rating systems.

Furthermore, as a credence product, the quality of medical services is difficult for patients to evaluate, even after treatments [13,14], so later patients may doubt whether former patients have clear evaluations of their physicians and treatments. In particular, different patients have different opinions about how good a physician should be to be rated with 5 stars and how bad a physician should be to be rated with 1 star. Besides the credence traits of medical services, web-based ratings influence physicians’ reputation and rankings on web-based review platforms, so physicians may be averse to receiving low ratings. Patients always need to build enduring relationships with their physicians [8], so dissatisfied patients may be afraid of being treated worse if they provide low scores for their physicians [15]. To help build physicians’ web-based reputation, patients may give their physicians high scores to avoid lowering their physicians’ web-based ratings. They may only express complaints in review texts, resulting in ambivalence, such as conflicting feelings, beliefs, and reactions toward physicians. Because of ambivalent reviews, later patients will consider whether physicians manipulate web-based ratings to obtain higher rankings and reputations by asking their patients to rate them higher [16]. Medical services mostly depend on direct contact between physicians and patients, which is unlike the web-based shopping context where sellers and consumers make indirect contact through products. Thus, web-based rating platforms for medical services may face more ambivalence than platforms for search or experience goods.

Owing to the aforementioned concerns in the health medical service context, the concentration of web-based ratings may be more serious, and whether patients who give 5-star ratings are truly satisfied with their physicians is still unclear. These phenomena seriously harm the helpfulness of web-based reviews and review systems. As 2 inseparable parts of a web-based review, review content and the corresponding numerical rating play decisive roles in determining review helpfulness. Schlosser [17] confirmed the importance of considering both qualitative (eg, the text) and quantitative (eg, the numerical rating) aspects in the evaluation of a review. Regarding the aforementioned issues, the relationship between review rating and content may substantially influence review helpfulness.

To improve the helpfulness of web-based review platforms, this study explored whether the ambivalence between rating and review sentiment influences the helpfulness of a review. If so, what other factors may influence this relationship? The remainder of this paper is organized as follows. In the *Literature Review* section, we report on a literature review of web-based review helpfulness and ambivalence to identify the research gaps. Our hypotheses and research model are proposed in section 3. The empirical test and results are described in section 4. Sections 5 and 6 discuss the results, theoretical contributions, and practical implications.

### Literature Review

#### The Determinants of Web-Based Reviews’ Helpfulness

The helpfulness of web-based reviews is defined as consumers’ perceived value of web-based reviews while making purchase decisions [18], which is the review readers’ perceptions rather than actual helpfulness. Hong et al [19] summarized the major determinants of review helpfulness, including review-related factors (eg, review depth, rating, and review age) and reviewer-related factors (eg, reviewer information disclosure and expertise). In this study, we focused only on review-related factors.

Review-related factors can be classified into 2 parts: review contents and numerical ratings. In most cases, review contents containing more information are more helpful because readers can learn more about the targets. This is demonstrated in several aspects, such as review depth or length [18,20-22], review with both pros and cons [17,23], and information quality [24]. The emotions expressed in review contents were also studied. Review sentiment enhances review helpfulness [20], and both negative emotions [25,26] and positive emotions [27] were found to be useful.

The numerical rating and content are inseparable. For most review platforms, numerical ratings range from 1 to 5 stars. Cao et al [23] and Choi and Leon [22] found that reviews with extreme ratings (eg, 1 star and 5 stars) are more helpful than those with neutral ratings, but which extreme rating is more helpful is controversial. Eslami et al [21] and Chua and Banerjee [28] found that lower ratings are more helpful, but Quaschning et al [29] found that positive ratings are more helpful. The quadratic review rating was also studied [18], but this nonlinear relationship was not substantial in Hong et al’s [19] meta-analysis.

To better explain the aforementioned inconsistent conclusions, the interaction between review content and numerical rating was considered. Reviews with 2-sided arguments are more helpful when the rating is moderately favorable [17], and extremely negative ratings are more helpful when the average rating is high [30]. Product type (search vs experience) is another factor that influences the conclusions. Extreme ratings are less useful than moderate ratings for experiential goods [18], and product intangibility moderates the effect of review extremity and depth on review helpfulness [22].

#### The Ambivalence in Web-Based Reviews

In conflict theory, ambivalence is defined as the result of a particular configuration of response alternatives, and response alternatives should have contradictory implications with subjectively equal importance [31]. Two types of ambivalence on web-based review platforms were investigated in existing research.

The first ambivalence is the inconsistency between an individual review and the aggregated review of a product (eg, a single rating of a product is 1 star, but the average rating of the product is 5 stars). Existing studies (eg, the studies by Choi and Leon [22], Gao et al [32], Qiu et al [33], and Cao et al [34]) focused on this and defined this as “conflicting ratings” or “inconsistent reviews.” Conflicting ratings decrease the credibility and diagnosticity of reviews because they reduce later consumers’ product-related attributions, and this impact is more salient for positive reviews [33]. Information that conflicts with individuals’ prior beliefs is perceived as less credible and helpful, so conflicting ratings are less helpful [22]. However, Aghakhani et al [35] found that conflicting ratings enhance review helpfulness because of negativity bias. The aforementioned studies consider the ambivalence between individual ratings and aggregated ratings, but consumers also read each review rather than only relying on summary statistics [36], so we intended to focus on each review.

The second type of ambivalence focuses on a single review, including ambivalence caused by opinions or sentiments. Schlosser [17] found that reviews with both pros and cons of products are less useful than 1-sided reviews unless the ratings are moderate. Web-based reviews with higher title-content similarity are more helpful because repeated exposure to a stimulus can enhance individuals’ preferences for the content [37]. The inconsistency between a review text and its attendant rating decreases review helpfulness because it leads to greater cognitive costs for later consumers [35]. However, Aghakhani et al’s [35] measure of inconsistency is based on human coders’ perception, which is also from the perspective of review readers. The evaluative-cognitive consistency theory implies that individuals are not always consistent, but they expect others to be consistent [17], so reviewers and review readers may have different perceptions about which reviews are inconsistent. Just telling reviewers to write consistent reviews may not be useful, and there is a need to further investigate the cause of the consistency, which platforms can improve.

#### Summary of Literature

In summary, the determinants of web-based review helpfulness have been studied comprehensively and thoroughly in the existing literature, but some gaps have not yet been well addressed. The first is related to the influence of the review target. Search and experience products (eg, books, smartphones, movies, and hotels) were widely studied, but attention to credence products and services, such as medical services, was rare. The quality of credence products is difficult to evaluate even after consumption [13,14], so former consumers also have no clear evaluations. In terms of medical services, Gao et al [8] found that the web-based ratings of physicians have positive relationships with offline patients’ perceptions of physicians’ quality; however, because of the credence traits of medical services, Saifee et al [38] found no substantial relationship between the web-based reviews of physicians and their clinical outcomes. On a review platform in China, 88% and 91% of ratings were positive for physicians’ treatment and bedside manner, respectively, even though the reviewers were anonymous [39]. Such concentrated ratings make it difficult to distinguish between good and bad physicians. Therefore, there is a need to explore whether the web-based reviews of physicians are helpful and what factors can influence review helpfulness.

The second gap is related to the interaction between the numerical rating and review content. Numerical ratings are widely used to measure reviewers’ attitudes [18,40], and later consumers always use ratings to judge the quality of products [26], so there is an assumption that both reviewers and readers believe that 5-star ratings should be combined with totally positive sentiments in texts and 1-star ratings with totally negative sentiments. This is important for existing studies, but to the best of our knowledge, there is no strong evidence for this assumption. Valdivia et al [41] suggested that ratings should not be used as labels of sentiments for web-based reviews because reviewers tend to rate positively but write negatively, and vice versa. The consistency between numerical ratings and consumers’ attitudes may not always be true [42,43] because consumers may have different opinions about what extent of satisfaction or dissatisfaction warrants a 5-star or 1-star rating, respectively.

Owing to inconsistent review sentiments and ratings, review readers may feel confused about why the reviewers gave 5-star ratings with some negative feelings, and the ambivalence may also lead readers to suspect that the reviews are false [17] or think that the reviewers are not serious. More importantly, the quality and usefulness of web-based review systems are reduced if the numerical ratings do not fit the review contents [44]. Readers also need to spend more time and effort judging whether they should rely on the rating or text and analyze which information is true [25,35]. These phenomena are not beneficial to the usefulness and development of web-based review systems. Therefore, there is a need to further investigate the relationship between reviewers’ emotions expressed in review content and their numerical rating, as well as its impact on web-based review helpfulness.

### Theoretical Background and Hypotheses Development

#### Ambivalent Attitudes in Web-Based Reviews

Ambivalent attitudes are defined as conflicting feelings, beliefs, and reactions toward a target [45]. That is, individuals simultaneously evaluate a target both positively and negatively [45], so the structure of ambivalent attitudes is inconsistent [46], leading to less persuasion [43].

Attitudes have 3 dimensions, and psychologists have proposed a tripartite model of attitudes [47]. The cognitive dimension of attitudes refers to individuals’ beliefs and thoughts about the targets, the affective dimension refers to the emotions and feelings of the targets [43], and the behavioral dimension refers to individuals’ past behaviors and future intentions regarding the targets [48]. After receiving treatment, patients can use web-based reviews to describe their experiences and express their attitudes toward physicians. A complete review of physicians includes numerical ratings and review texts [49], and the ambivalence between numerical ratings and review texts may influence review helpfulness. Numerical ratings and review texts can be conceptualized as the cognitive and affective dimensions of attitudes, respectively. Besides, posting web-based reviews is a post hoc behavior that is exhibited after patients receive treatment from physicians, so we do not consider the behavioral dimension of attitudes.

Pan et al’s [50] study on technological change stated that the cognitive dimension of attitudes focuses on the functions of information systems, such as update frequency [51], perceived usefulness [52], perceived ease of use [52], and social influence [52]. The affective dimension is operationalized as satisfaction [51,52] and comfort with technological change [52]. Pan et al [50] measured the cognitive dimension of users’ attitudes through their perception of their internet-based participation in technological changes. According to the aforementioned studies, the cognitive dimension should focus on the objective function of, efficiency of, and experience with the targets. In the context of web-based reviews of physicians, numerical ratings are patients’ quantitative evaluations of physicians and treatments. For example, on RateMDs, patients can evaluate their physicians on 4 aspects, including helpfulness, punctuality, staff, and knowledge; on Vitals, patients can evaluate their wait time, ease of making appointments, staff, diagnosis, etc. On the basis of the definition of the cognitive dimension of attitudes [43] and Pan et al’s [50] summary of its measurements, the numerical ratings of physicians was used to measure reviewers’ cognitive dimension of patients’ attitudes toward physicians’ treatments because different aspects of the physicians and their treatments can be evaluated through the scores. Review texts can express patients’ feelings and emotions toward the physicians and their treatments, so positive or negative emotions expressed in texts can measure the affective dimension of reviewers’ attitudes.

Because the tripartite model separates attitudes into different dimensions, attitudes can be ambivalent [31]. Therefore, ambivalence exists in a single review because of the inconsistency between the numerical rating, which reflects the reviewer’s cognitive attitude, and the textual content, which expresses the reviewer’s affective attitude. However, this ambivalence is aversive [45]. Ambivalent attitudes are regarded as weak attitudes [45], and they reduce persuasion [43]. Perceived equivocality in texts also decreases the quality of consumers’ decisions [53]. Therefore, when conflicting rating and emotion are expressed in a review, the review readers may be confused about the reviewer’s attitude and even doubt the truth [25]. When reviews have ambivalent attitudes, later patients may be confused about the reviewers’ attitudes, and they need to spend more time and effort judging the credibility and reliability of the reviews [35]. Therefore, ambivalence may harm the helpfulness of reviews.

#### Risk Reduction Perspective and Ambivalent Attitudes

Although ambivalence is expected to reduce review helpfulness, this effect is unlikely to be the same for all reviews because from the risk reduction perspective, being clear about the possible risks of a decision in advance is helpful for review readers.

Risk reduction is the major motivation for later consumers to interpret web-based reviews with care [54]. The theory of risk taking by Sheth and Venkatesan [55] states that consumers always purchase products under uncertainty, and seeking opinions from other similar buyers is the major way to reduce the uncertainty. Thus, web-based reviews with some possible negative consequences (eg, physicians without good attitudes) that patients will face are helpful in reducing risks. Besides risk reduction, to confirm choices, having selected a product, customers need reassurance that they have made good choices [54,56]. In our research context, the helpfulness of physicians’ web-based reviews is defined as later patients’ perceived value of the reviews before choosing physicians. When patients intend to choose a physician, they read web-based reviews to confirm that their choice is correct. Therefore, to reduce risks, low cognitive attitude scores may be helpful for later patients in identifying the possible risks of their choices.

However, this positive effect may vary from one review to another; based on the existing literature, we introduce review valence to better understand the different mechanisms. Review valence is defined as the reviewers’ positive or negative sentiments and emotions expressed in reviews [29,57]. For positive reviews, if the weaknesses of the physician are declared in the cognitive dimension of the reviews, later patients’ risk of choosing the physician can be reduced. For example, on RateMDs, if a patient is sensitive to waiting time, he can avoid physicians with low scores on punctuality. Therefore, from the risk reduction perspective, positive valence reviews with the drawbacks of physicians may be more helpful than reviews just praising physicians, leading to hypothesis 1 (H1): for positive valence web-based reviews, the ambivalence of the affective and cognitive dimensions of attitudes enhances the helpfulness of the reviews.

However, this effect may be different for negative valence reviews. If previous patients give high scores for physicians and their treatments, they should be satisfied with the physicians, so why they express negative feelings toward the physicians may confuse later patients. Therefore, for negative valence reviews, ambivalence may reduce the helpfulness of the reviews because it reduces persuasion [43], and later patients may spend more cognitive effort evaluating the information [35], leading to hypothesis 2 (H2): for negative valence web-based reviews, the ambivalence of the affective and cognitive dimensions of attitudes weakens the helpfulness of the reviews.

**Figure 1:**
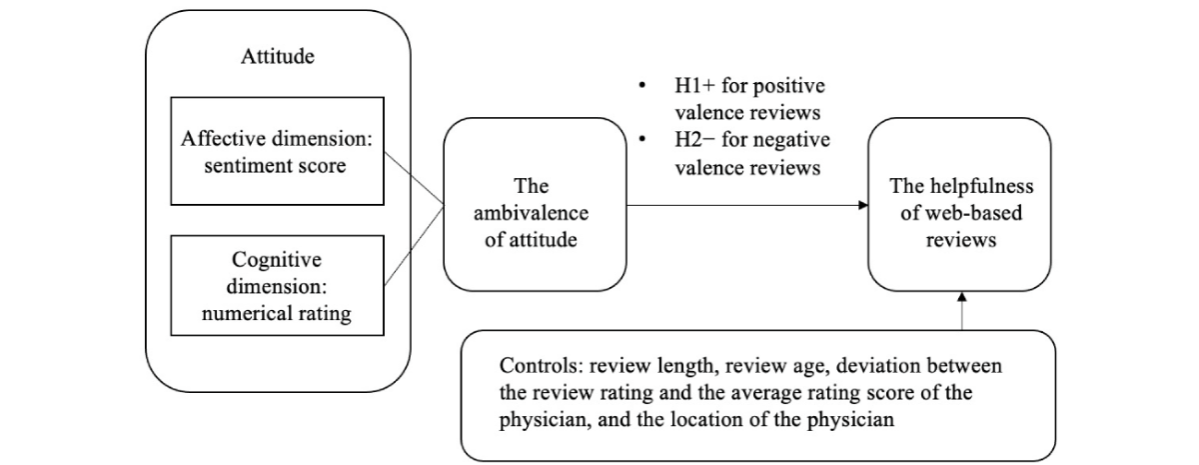
The research model. H1: hypothesis 1; H2: hypothesis 2.

The research model is depicted in Figure 1.

## Methods

### Data Collection

**Figure 2:**
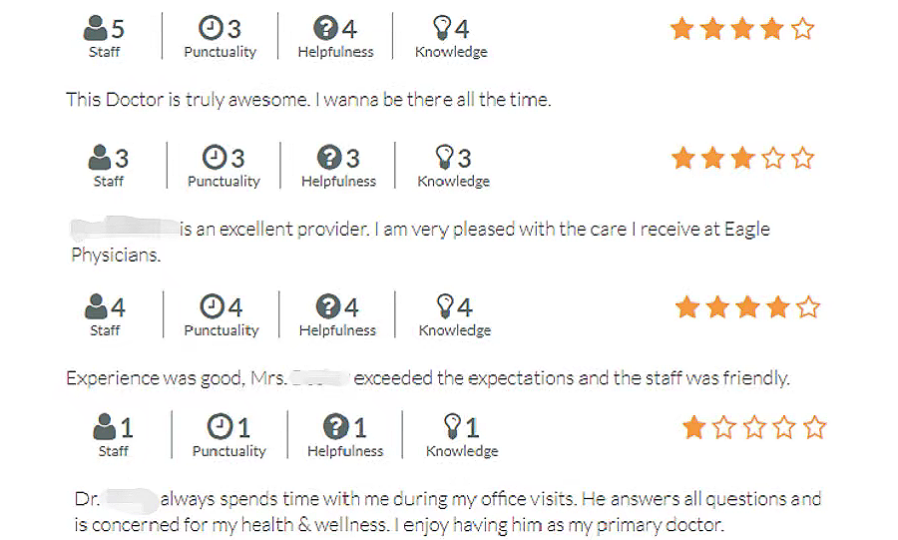
Some examples of web-based reviews on RateMDs.

We used Python to develop a web crawler to collect data from one of the largest physician review websites (RateMDs) in May 2019. This website is anonymous for reviewers, so we could exclude the impact of reviewer-related factors on the helpfulness of reviews. This website also provides details of each review, such as ratings for 4 aspects of physicians’ treatments and when the reviews were posted. Figure 2 presents several examples. Our sample included reviews of the top 5000 family physicians and general practitioners on the website. Some non-English reviews were excluded, and the final sample included 114,378 reviews with ratings for 3906 physicians.

Pan et al [50] stated that the function or use experience of an information system is used to measure the cognitive dimension of an attitude. On this website, 4 different dimensions (eg, helpfulness, staff, knowledge, and punctuality) of physicians’ treatments are evaluated by numerical ratings (1-5), and based on the definition of cognitive attitude [43], the numerical rating of physicians can be used to measure reviewers’ thoughts and beliefs about the physicians’ treatments because the different dimensions of the physicians and their treatments can be evaluated through the scores. We obtained the average score of each review as the cognitive dimension of an attitude for each review.

Reviewers also write texts to express their feelings about physicians’ services; we used SentiStrength by Khan [58] to calculate the sentiment score of the review text, which was considered the affective dimension of the corresponding reviewer's attitude [59]. SentiStrength has been widely used in many studies [60], and it is a desirable tool with better performance than other general machine learning approaches [61] used to estimate the strength of positive and negative sentiments in short texts. SentiStrength calculates the sentiment strength of each word in a text and provides both a positive strength score (*positiveScore*) and a negative sentiment strength score (*negativeScore*) of the text, ranging from 1 (not positive) to 5 (extremely positive) and −1 (not negative) to −5 (extremely negative), respectively. After obtaining the strengths of the text, we referred to the following formula by Chen et al [60] to generate the sentiment score (*sentScore*) of each review. Sentiment scores range from −4 (extremely negative) to 4 (extremely positive), and 0 indicates that the review text has a neutral sentiment.


*sentScore_i_ = positiveScore_i_ + negativeScore_i_*
**(1)**

### Variables and Operationalization

To test our research model, we followed previous studies to operationalize the constructs. Using the secondary data collected from review websites, review helpfulness was mostly measured by the number of helpful votes (such as in the studies by Mudambi and Schuff [18], Cao et al [23], and Filieri et al [30]), percentage of helpful votes (such as in the studies by Schlosser [17] and Choi and Leon [22]), and probability that a review receives helpful votes (such as in the study by Pan and Zhang [62]). The number of helpfulness votes can be used to directly measure review readers’ helpfulness perceptions. As the website does not provide the percentage of helpful votes, we used the number of helpful votes (*helpfulNum*) and probability of being rated as helpful to measure review helpfulness.

**Figure 3:**
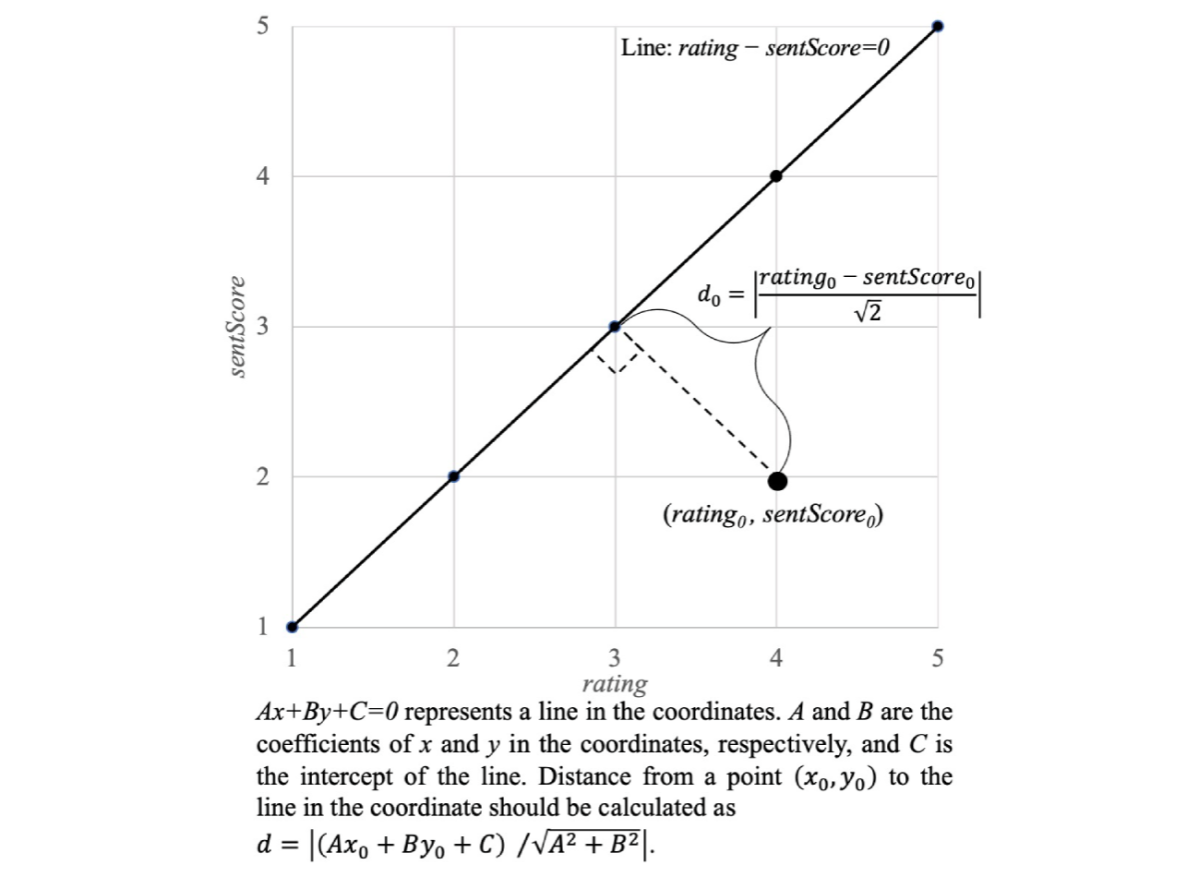
The line of consistent numerical rating and sentiment score.

The independent variable was the degree of ambivalence (*ambivalence*) between the affective and cognitive dimensions of attitude in a single review, which was calculated using the sentiment score (*sentScore*) and numerical rating (*rating*). Because the rating on the review website ranges from 1 to 5, we changed the range of sentiment scores from −4 to 4 to 1 to 5 through a linear transformation. According to the premise of rating and sentiment [18], reviews with consistent ratings and sentiment scores should satisfy *sentScore_i_=rating_i_*, where the subscript *i* indicates the index of a review. For example, *sentScore_i_=rating_i_* when *sentScore_i_=3* and the *rating_i_=3* and when *sentScore_i_=4* and the *rating_i_=4*. By contrast, when the sentiment score and rating are inconsistent, the reviews are ambivalent. For example, the reviews are ambivalent when *sentScore_i_=5* and the *rating_i_=1*, which is similar to the fourth example review shown in Figure 2. Thus, the rating and emotion scores of consistent reviews are distributed on the line in Figure 3. Therefore, we considered the distance of each point determined by the x-axis (*rating*) and y-axis (sentScore) to the line in Figure 3 as the degree of ambivalence. Following the formula of the distance from a point to the line, for reviews with inconsistent attitudes, the degree of ambivalence was the distance between the point (*rating_0_*, *sentScore_0_*) and the consistent line (*rating-sentScore=0*), which was calculated as *d=|(Ax_0_+By_0_+C/√(A^2^+B^2^))|* (see the explanation in Figure 3); therefore, the degree of the ambivalence between affective and cognitive attitudes in our model was calculated using formula 2, and the ambivalence was calculated as the square of the degree of the ambivalence (formula 3).


*d_i_ = |(sentScore_i_-rating_i_) / √2|*
**(2)**

*ambivalence_i_ = d^2^_i_ = (sentScore_i_ − rating_i_)^2^ / 2*
**(3)**

To test our hypotheses, we classified our samples into positive and negative valence groups according to review valence. Numerical ratings were mostly used in previous literature to indicate the valence of reviews (eg, the studies by Quaschning et al [29] and Pan and Zhang [62]). However, according to Valdivia et al [41], ratings should not be used as sentiment labels for web-based reviews because reviewers tend to rate positively but write negatively; therefore, we measured the valence of a review as a binary variable to indicate the positive or negative emotion of the reviews. The emotion score was calculated using SentiStrength. When the positive score was higher than the negative score, the review valence was positive; conversely, when the negative score was higher than the positive score, the review valence was negative. To better explain the results, we marked the reviews with the same negative score and positive score as neutral valence subsamples. Finally, 114,378 reviews are in our sample, 83,223 (72.76%) reviews had a positive valence, 14,814 (12.95%) reviews had a negative valence, and 16,341 (14.29%) reviews were neutral.

Hong et al’s [19] meta-analysis stated that review-related factors, such as review length and review age, can enhance review helpfulness. Reviews with more words have more in-depth information and are more helpful [19], and more readers may read reviews posted a long time ago. Therefore, we included review length (*length*) and the total months after a review was posted (*months*) as 2 control variables. The difference between the review rating and average rating score of the physician (*devAvgRating*) [22] was also controlled because the existing literature has confirmed the impact of inconsistency between an individual rating and the aggregated rating of a product on review helpfulness. The state (*state*) where the physician was located was also controlled to exclude regional differences.

The data descriptions are presented in Table 1.

**Table 1:** The data descriptions.

Variable name		Descriptions	Type
**Dependent variable**			
	helpfulNum	The total number of helpful votes of a review	Numeric
**Independent variable**			
	ambivalence	The inconsistency between the affective and cognitive components of an attitude, which is calculated as equation (3)	Numeric
**Control variable**			
	length	The number of words in a review	Numeric
	months	The total number of months after a review was posted on the web	Numeric
	devAvgRating	The absolute value of deviation between the review rating and the average rating score of the physician	Numeric
	state	The state where the physician is located	Dummy

### Ethical Considerations

As the data involved in this study were collected from the internet, no experiment or manipulation was conducted on humans, animals, and other creatures. Hence, ethics approval is not applicable for this study.

## Results

### Descriptive Statistics

The summary and correlation matrix are presented in Table 2. The absolute values of the correlations were all <0.2 and all *vif*<10, excluding multicollinearity [63].

**Table 2:** Summary and correlation matrix of variables.

	Value, mean (SD; range)	VIF^a^	1	2	3	4	5	6
helpfulNum	0.601 (1.798; 0-68)	—^b^	1	—	—	—	—	—
ambivalence	0.878 (0.910; 0-8)	1.04	0.031	1	—	—	—	—
length	48.646 (36.411; 1-189)	1.07	0.065	0.177	1	—	—	—
months	66.895 (47.505; 1-170)	1.02	−0.138	−0.040	0.111	1	—	—
devAvgRating	0.490 (0.638; 0-5)	1.02	0.115	−0.059	0.118	−0.027	1	—
state	8.007 (7.549; 1-67)	1.00	0.016	0.019	−0.018	−0.003^c^	−0.020	1

^a^VIF: variance inflation factor,

^b^Not applicable.

^c^The *P* value of this correlation coefficient was .88. Except for this correlation, all correlation coefficients in the table had *P* values <.001.

### Statistical Analysis: Ordinary Least Squares

First, using the 3 subsamples we classified based on review valence (eg, positive, negative, and neutral), we used the ordinary least squares (OLS) regression to test our hypotheses. The regression model is shown in formula 4, where *i* is the index of reviews, *β_0_* is the constant of the model, *β_1_* is the coefficient of the independent variable, *β_2_* to *β_5_* are the coefficients of the control variable, and *μ* is the error term.


*helpfulNum_i_ =*
*β_0_ +*
*β_1_ambivalence_i_ +*
*β_2_length_i_ + β_3_months_i_ +*
*β_4_devAvgRating_i_ +*
*β_5_state_i_ + μ*
**(4)**

We used Stata (version 15.1; StataCorp) to obtain the OLS results. The results are presented hierarchically in Table 3. For the 3 subsamples, model 1 included only control variables, and then the independent variable was introduced in model 2. The first column under model 2 shows that for positive valence reviews, the ambivalence between the cognitive and affective dimensions of attitudes increases the helpfulness of the reviews (β_positive 1_=.046; *P*<.001), indicating that as the ambivalent attitude in a single review increases by 1 unit, the helpfulness of the review increases by 0.046, supporting H1. The third column under model 2 shows that for negative valence reviews, ambivalence can decrease the helpfulness of the reviews (β_negative 1_=−.059; *P*=.002), indicating that as the ambivalent attitude in a single review increases by 1 unit, the helpfulness of the review decreases by 0.059, supporting H2. However, the second column of model 2 shows that for neutral reviews, the effect is not significant (β_neutral 1_=−.030; *P*=.23), indicating that ambivalence has no significant impact on helpfulness.

**Table 3:** The results of ordinary least squares (dependent variable: the number of helpful votes of a review).

Variable				Model 1^a^							Model 2^b^					
				1: positive subsample		2: neutral subsample		3: negative subsample		1: positive subsample			2: neutral subsample		3: negative subsample	
**ambivalence**																
	β_1_			—^c^		—		—		.046			−.030		−.059	
	*P* value			—		—		—		<.001			.23		.002	
	SD			—		—		—		0.013			0.025		0.019	
**length**																
	β_2_			.003		.004		.003		.003			.004		.003	
	*P* value			<.001		<.001		<.001		<.001			<.001		<.001	
	SD			0.0002		0.0004		0.0005		0.0002			0.0004		0.0005	
**months**																
	β_3_			−.005		−.007		−.006		−.005			−.007		−.007	
	*P* value			<.001		<.001		<.001		<.001			<.001		<.001	
	SD			0.0001		0.0003		0.0005		0.0001			0.0003		0.0005	
**devAvgRating**																
	β_4_			.248		.178		.254		.241			.168		.193	
	*P* value			<.001		<.001		<.001		<.001			<.001		<.001	
	SD			0.013		0.020		0.019		0.013			0.022		0.027	
**state (controlled): constant term**																
	β_5_		.019		.039		.051		−.006			.100		.261		
	*P* value		.53		.68		.77		.85			.35		.15		
	SD		0.030		0.094		0.170		0.032			0.107		0.182		

^a^Model 1: positive subsample, adjusted *R^2^=*0.0499, *F*_69_=64.39, n=83,223 (72.76%); neutral subsample, adjusted *R^2^=*0.0599, *F*_65_=16.08, n=16,341 (14.29%); negative subsample, adjusted *R^2^=*0.1471, *F*_65_=39.15, n=14,814 (12.95%).

^b^Model 2: positive subsample, adjusted *R^2^=*0.0501, *F*_70_=63.66, n=83,223 (72.76%); neutral subsample, adjusted *R^2^=*0.0599, *F*_66_=15.87, n=16,341 (14.29%); negative subsample, adjusted *R^2^=*0.1477, *F*_66_=38.74, n=14,814 (12.95%).

^c^Model 1 was the benchmark for model 2.

The aforementioned results show that if a review has a positive attitude toward the physician, the inconsistency between the sentiment score and numerical rating can increase the helpfulness of the review. For example, if the patient feels good about the physician and writes the review content such that it shows that they are satisfied with the physician but gives a low numerical rating to point out the drawbacks of the physician, such as that the physician is not punctual or that the physician’s attitude is not good, the helpfulness of the review increases. However, if a review has a negative attitude toward the physician, the inconsistency between the sentiment score and numerical rating can decrease the helpfulness of the review. For example, if the patients writes the review content such that it shows that they are not satisfied with the physician but gives the physician a high numerical rating (eg, 5 stars), the helpfulness of the review decreases.

### Statistical Analysis: Logistic Regression

Then, we conducted logistic regressions (formula 5) to further understand the probability of being rated as helpful. In formula 5, *p* is the probability that a review is voted as helpful (*helpfulNum*>0) and the left side of the equal sign is the logarithm of the probability, *θ_0_* is the constant of the model, *θ_1_* is the coefficient of the independent variable, *θ_2_* to *θ_5_* are the coefficients of the control variables, and *ω* is the error term of the model.

*ln[p / (1* − *p)] =*
*θ_0_ +*
*θ_1_ambivalence_i_ +*
*θ_2_length_i_*
*+ θ_3_months_i_ + θ_4_devAvgRating_i_ +*
*θ_5_state_i_ + ω* **(5)**

The results of the logistic regressions are listed hierarchically in Table 4. The first column under model 4 shows that for positive valence reviews, the ambivalence between the cognitive and affective dimensions of attitudes can increase the helpfulness of the reviews (θ_positive 1_=0.056; *P*=.005). As *ambivalence* increases by 1 unit, the probability of being voted as helpful increases by 5.8% (*exp^0.056^*=1.058), supporting H1. The third column under model 4 shows that for negative valence reviews, ambivalence can decrease the helpfulness of the reviews (θ_negative 1_=−0.080; *P*<.001). As *ambivalence* increases by 1 unit, the probability of being voted as helpful decreases by 8.3% (*exp^0.080^*=1.083), supporting H2. The second column under model 4 shows that for neutral valence reviews, ambivalence can decrease the helpfulness of the reviews (θ_neutral 1_=−0.060; *P*=.03). As *ambivalence* increases by 1 unit, the probability of being voted as helpful decreases by 6.2% (*exp^0.060^*)=1.062.

**Table 4:** The results of the logistic regressions.

Variable				Model 3^a^						Model 4^b^				
				1: positive subsample		2: neutral subsample		3: negative subsample		1: positive subsample		2: neutral subsample		3: negative subsample
**ambivalence**														
	𝜃_1_			—^c^		—		—		0.056		−0.060		−0.080
	*P* value			—		—		—		.005		.03		<.001
	SD			—		—		—		0.020		0.028		0.016
**length**														
	𝜃_2_			0.006		0.007		0.005		0.006		0.007		0.005
	*P* value			<.001		<.001		<.001		<.001		<.001		<.001
	SD			0.0003		0.0004		0.0004		0.0003		0.0004		0.0004
**months**														
	𝜃_3_			−0.010		−0.010		−0.009		−0.010		−0.011		−0.009
	*P* value			<.001		<.001		<.001		<.001		<.001		<.001
	SD			0.0002		0.0004		0.0004		0.0002		0.0004		0.0004
**devAvgRating**														
	𝜃_4_			0.277		0.238		0.268		0.267		0.220		0.187
	*P* value			<.001		<.001		<.001		<.001		<.001		<.001
	SD			0.019		0.023		0.016		0.019		0.024		0.023
**state (controlled): constant term**														
	𝜃_5_		−3.213		−3.104		−2.468		−3.244		−2.982		−2.182	
	*P* value		<.001		<.001		<.001		<.001		<.001		<.001	
	SD		0.100		0.226		0.237		0.101		0.232		0.244	

^a^Model 3: positive subsample, Wald χ^2^_63_=4635.7, *P*<.001, pseudo *R*^2^=0.0540, n=83,160 (72.76%); neutral subsample, Wald χ^2^_58_=1219.9, *P*<.001, pseudo *R*^2^=0.0688, n=16,300 (14.29%); negative subsample, Wald χ^2^_55_=1084.7, *P*<.001, pseudo *R*^2^=0.0619, n=14,780 (12.95%). Some samples were dropped because for physicians in some states, no reviews received helpful votes, and Stata dropped these observations.

^b^Model 3: positive subsample, Wald χ^2^_64_=4638.4, *P*<.001, pseudo *R*^2^=0.0541, n=83,160 (72.76%); neutral subsample, Wald χ^2^_59_=1221.6, *P*<.001, pseudo *R*^2^=0.0690, n=16,300 (14.29%); negative subsample, Wald χ^2^_56_=1108.7, *P*<.001, pseudo *R*^2^=0.0631, n=14,780 (12.95%). Some samples were dropped because for physicians in some states, no reviews received helpful votes, and Stata dropped these observations.

^c^Model 3 was the benchmark for model 4.

### Statistical Analysis: Tobit Regression

Finally, considering that the number of helpful votes is a censored sample and that readers cannot mark a review as “very useful” or “not very useful,” we could not know the degree of helpfulness [18]. Besides, the mean value of our sample with *usefulNum*>0 was 2.120 (n=32,448, SD 2.859), which was much higher than the mean of all the samples (0.601; N=114,378, SD 1.798). Thus, to eliminate the bias of OLS, we used the Tobit model to better understand the results. The basic Tobit model is shown in formula 6, where *i* indicates the index of reviews, *usfulNum^a^_i_* is the latent dependent variable that is censored by 0, *α_0_* is the constant of the model, *α_1_* is the coefficient of the independent variable, *α_2_* to *α_5_* are the coefficients of the control variables, and *ε* is the error term of the model.


*usefulNum^a^_i_ = α_0_ + α_1_ambivalence_i_ + α_2_length_i_ + α_3_months_i_ + α_4_devAvgRating_i_ + α_5_state_i_ + ε*
**(6)**

The results are listed hierarchically in Tables 5-7 based on the 3 subsamples respectively.

**Table 5:** The results of the Tobit regression for positive valence reviews (dependent variable: the number of helpful votes of a review).

Variable			Model 5: positive valence (n=83,223)							Model 6: positive valence (n=83,223)				
			1: Tobit (y^a^)		2: censored sample (y^a^|y>0)		3: truncated sample (y|y>0)		1: Tobit (y^a^)			2: censored sample (y^a^|y>0)		3: truncated sample (y|y>0)
**ambivalence**														
	α_1_	—^b^		—		—		.131			.030		.031	
	*P* value	—		—		—		.001			.004		.002	
	SD	—		—		—		0.041			0.010		0.010	
**length**														
	α_2_	.012		.003		.003		.012			.003		.003	
	*P* value	<.001		<.001		<.001		<.001			<.001		<.001	
	SD	0.001		0.0004		0.0002		0.001			0.0004		0.0002	
**months**														
	α_3_	−.022		−.005		−.005		−.022			−.005		−.005	
	*P* value	<.001		<.001		<.001		<.001			<.001		<.001	
	SD	0.0004		0.001		0.004		0.0004			0.001		0.0004	
**devAvgRating**														
	α_4_	.712		.164		.166		.688			.158		.160	
	*P* value	<.001		<.001		<.001		<.001			<.001		<.001	
	SD	0.039		0.026		0.014		0.040			0.025		0.014	
**state (controlled): constant**														
	α_5_	−6.536		—		—		−6.608			—		—	
	*P* value	<.001		—		—		<.001			—		—	
	SD	0.170		—		—		0.172			—		—	
Log-likelihood			−83,597.9		—		—		−83,592.9			—		—
LR^c^ chi-square (*df*)			6103.5 (69)		—		—		6113.6 (70)			—		—
*P* value			<.001		—		—		<.001			—		—
Pseudo *R*^2^			0.0352		—		—		0.0353			—		—

^a^The y^a^ is the latent variable because *helpfulNum* is censored by zero.

^b^Model 5 was the benchmark for model 6.

^c^LR: likelihood ratio test.

**Table 6:** The results of the Tobit regression for neutral valence reviews (dependent variable: the number of helpful votes of a review).

					Model 7: neutral valence (n=16,341)										Model 8: neutral valence (n=16,341)						
					1: Tobit (y^a^)			2: censored sample (y^a^|y>0)			3: truncated sample (y|y>0)			1: Tobit (y^a^)				2: censored sample (y^a^|y>0)			3: truncated sample (y|y>0)
**ambivalence**																					
	α_1_			—^b^			—			—			−.142				−.038			−.035	
	*P* value			—			—			—			.03				.06			.04	
	SD			—			—			—			0.066				0.020			0.017	
**length**																					
	α_2_			.015			.004			.004			.015				.004			.004	
	*P* value			<.001			<.001			<.001			<.001				<.001			<.001	
	SD			0.001			0.001			0.005			0.001				0.001			0.0004	
**months**																					
	α_3_			−.027			−.007			−.007			−.027				−.007			−.007	
	*P* value			<.001			<.001			<.001			<.001				<.001			<.001	
	SD			0.001			0.002			0.001			0.001				0.002			0.001	
**devAvgRating**																					
	α_4_			.568			.151			.141			.523				.139			.130	
	*P* value			<.001			<.001			<.001			<.001				<.001			<.001	
	SD			0.052			0.038			0.021			0.056				0.036			0.020	
**state (controlled)**																					
	**Constant**																				
		α_5_	−7.060			—			—			−6.769				—			—		
		*P* value	<.001			—			—			<.001				—			—		
		SD	0.434			—			—			0.454				—			—		
Log-likelihood					−19,227.2			—			—			−19,224.9				—			—
LR^c^ chi-square (*df*)					1576.5 (69)			—			—			1581.1 (70)				—			—
*P* value					<.001			—			—			<.001				—			—
Pseudo *R*^2^					0.0394			—			—			0.0395				—			—

^a^The y^a^ is the latent variable because *helpfulNum* is censored by zero.

^b^Model 7 was the benchmark for model 8.

^c^LR: likelihood ratio test.

**Table 7:** The results of the Tobit regression for negative valence reviews (dependent variable: the number of helpful votes of a review).

					Model 9: negative valence (n=14,814)										Model 10: negative valence (n=14,814)						
					1: Tobit (y^a^)			2: censored sample (y^a^|y>0)			3: truncated sample (y|y>0)			1: Tobit (y^a^)				2: censored sample (y^a^|y>0)			3: truncated sample (y|y>0)
**ambivalence**																					
	α_1_			—^b^			—			—			−.217				−.072			−.061	
	*P* value			—			—			—			<.001				.005			<.001	
	SD			—			—			—			0.042				0.026			0.015	
**length**																					
	α_2_			.013			.004			.004			.012				.004			.003	
	*P* value			<.001			<.001			<.001			<.001				.001			<.001	
	SD			0.001			0.001			0.001			0.001				0.001			0.001	
**months**																					
	α_3_			−.024			−.008			−.007			−.024				−.008			−.007	
	*P* value			<.001			<.001			<.001			<.001				.001			<.001	
	SD			0.001			0.002			0.001			0.001				0.002			0.001	
**devAvgRating**																					
	α_4_			.713			.237			.199			.496				.165			.139	
	*P* value			<.001			<.001			<.001			<.001				.002			<.001	
	SD			0.041			0.073			0.036			0.058				0.054			0.028	
**state (** **controlled)**																					
	**Constant**																				
		α_5_	−6.615			—			—			−.583				—			—		
		*P* value	<.001			—			—			<.001				—			—		
		SD	0.551			—			—			0.569				—			—		
Log-likelihood					−21,443.8			—			—			−21,430.2				—			—
LR^c^ chi-square (*df*)					1921.4 (67)			—			—			1948.6 (68)				—			—
*P* value					<.001			—			—			<.001				—			—
Pseudo *R*^2^					0.0429			—			—			0.0435				—			—

^a^The y^a^ is the latent variable because *helpfulNum* is censored by zero.

^b^Model 9 was the benchmark for model 10.

^c^LR: likelihood ratio test.

In the 3 tables, models 5, 7, and 9 contained only the control variables, and the independent variable was included in models 6, 8, and 10. The types of Tobit models show the results of the whole sample, censored sample, and truncated sample (*usefulNum>0*). The first column under each model shows the marginal effects for the latent variables, the second column under each model shows the marginal effects for the censored sample, and the third column under each model shows the marginal effects for the truncated sample.

For positive valence reviews (Table 5), all the coefficients of *ambivalence* in model 6 were significantly positive at *P*<.05, indicating that as the ambivalence of reviews increases, the reviews may become more useful for later patients. In model 6, the coefficient of the Tobit model indicated that as the ambivalence of reviews increases by 1 unit, there may be a latent increase in the helpfulness of the reviews by 0.131. The marginal effect of the censored sample indicated that as ambivalence increases by 1 unit, helpfulness increases by 0.030. The marginal effect of the truncated sample indicated that for all the reviews with *usefulNum>0*, as ambivalence increases by 1 unit, helpfulness increases by 0.031. Thus, the results support H1.

For negative valence reviews (Table 6), all the coefficients of *ambivalence* in model 10 were significantly negative at *P*<.05, indicating that as the ambivalence of reviews increases, the reviews will become less useful for later patients. The coefficient of the Tobit model indicated that as ambivalence increases by 1 unit, there may be a latent decrease in helpfulness by 0.217. The marginal effect of the censored sample indicated that as ambivalence increases by 1 unit, helpfulness decreases by 0.072. The marginal effect of the truncated sample indicated that for all the reviews with *usefulNum>0*, as ambivalence increases by 1 unit, helpfulness decreases by 0.061. Therefore, the results support H2.

For neutral valence reviews (Table 7), almost all the coefficients of *ambivalence* in model 8 were significantly negative at *P*<.05, indicating that as the ambivalent attitudes in reviews increase, the reviews may become less useful for later patients. The coefficient of the Tobit model indicated that as ambivalence increases by 1 unit, there may be a latent decrease in helpfulness by 0.142. The marginal effect of the censored sample indicated that as ambivalence increases by 1 unit, helpfulness decreases by 0.038. The marginal effect of the truncated sample indicated that for all reviews with *usefulNum*>0, as ambivalence increases by 1 unit, helpfulness decreases by 0.035.

## Discussion

### Principal Findings

Many studies have explored the impact of review- and reviewer-related factors on the helpfulness of web-based reviews [19], but they have mostly ignored whether the premise of consistency between ratings and sentiments is true. The existing literature (eg, the studies by Mudambi and Schuff [18], Choi and Leon [22], Cao et al [23], and Zhang et al [40]) and later review readers have always measured reviewers’ attitudes using numerical ratings, but based on the theory of ambivalent attitudes, reviewers may have ambivalent attitudes because attitudes have multiple dimensions, and ambivalence may occur among these dimensions [46]. The role of numerical ratings as sentiment labels should be reconsidered because reviewers tend to rate positively but write negatively, and vice versa [41]. Reviewers who rate physicians with 5 stars may not always be satisfied, and those who rate physicians with 1 star may have positive attitudes toward their physicians.

In this study, we found that for a positive valence review, the reviewer has a positive feeling toward the physician; however, total praise in the cognitive dimension of attitude is not helpful for later patients to evaluate the physician because from the risk reduction perspective, review readers want to reduce risks by reading web-based reviews [54]. For example, on RateMDs, if a patient is sensitive to waiting time, they can avoid physicians with low scores on punctuality. Thus, from the risk reduction perspective, positive valence reviews with the drawbacks of physicians may be more helpful than reviews just praising physicians. This phenomenon is also consistent with the reality that “no one is perfect.” Some weaknesses from a review are acceptable, and later patients may feel that these complex attitudes are more objective with more information about the shortcomings of a physician. Later patients will not be concerned with physicians’ manipulation of reviews, and the risks of their choices can be reduced, leading to a higher level of helpfulness.

However, for negative and neutral valence reviews, ambivalent attitudes are not useful, and later patients may feel confused about why the reviewers have bad feelings toward the physicians but rate them 5 stars. Besides, this ambivalence may lead to concern about whether reviewers give high ratings because they are afraid of their physicians, as low ratings can aggregate to negatively influence their physicians’ web-based ranks and reputations. When reviewers have bad feelings about a physician, the indication of the shortcomings of the physician in numeral ratings will be helpful for later patients, and complaints without low ratings for the services are not useful. Therefore, expressing the shortcomings of physicians’ services in web-based reviews are always useful.

### Theoretical Contributions

Considering the credence traits of medical services, this study explored the helpfulness of web-based reviews in the health care context. Although it is difficult to distinguish excellent physicians from ordinary physicians because web-based reviews of physicians concentrate on high ratings [1,3] and Saifee et al [38] found that there is no substantial relationship between physicians’ web-based reviews and clinical outcomes, this study found that web-based reviews are helpful for later patients in reducing risk, especially reviews that clearly state the shortcomings of physicians’ services.

Furthermore, this study contributes to the literature on web-based reviews by investigating the relationship between reviewers’ sentiments and numerical ratings. We found that the premise in previous studies [18,40] that 5-star ratings represent reviewers’ positive sentiments and 1-star ratings represent reviewers’ negative sentiments is not always true because reviewers’ attitudes have more than one dimension. Reviewers who give 5-star ratings to physicians may still have some complaints about their treatments, and reviewers who give 1-star ratings to physicians may praise their physicians. A similar phenomenon was also investigated by Aghakhani et al [35]; according to the tripartite model of attitudes [47], we further explored the cause of the ambivalence between reviewers’ emotions and numerical ratings. This study also confirms Valdivia et al’s [41] suggestion that review opinions, rather than ratings, should be used as the label of reviewers’ sentiments. Thus, web-based review research should address the use of numerical ratings as an indicator of reviewers’ attitudes.

Moreover, based on the tripartite model of attitudes and existing literature (eg, the study by Pan et al [50]), we contextualized the cognitive and affective dimensions of attitudes in the web-based review context using web-based ratings and sentiments extracted from review texts. The results of our study confirm the risk reduction role [54] of web-based reviews, even though ambivalent attitudes harm the helpfulness of web-based reviews. The results also confirm the negativity bias and further explain the mechanism of how negative information works in this research context. The “negativity bias” [25,26] in previous studies demonstrated that negative information (eg, the disadvantages of the products) always influences people more than positive information (eg, the advantages of the products) because humans are more sensitive to negative consequences and behave in a “risk-averse” manner [33]. Therefore, arguments with ambivalent attitudes may not always be useless because negative cognitive information about physicians is useful regardless of whether it is in positive or negative valence reviews, and later patients can obtain a comprehensive understanding of physicians in advance to minimize risks.

### Practical Implications

This study addresses an important issue in web-based review systems. We combined patients’ qualitative (ie, the sentiments about their physicians) and quantitative (ie, the numerical rating of their physicians) evaluations of their physicians, which can eliminate the intrinsic flaw of a single metric tool. This can directly improve web-based review systems in the health care context. Specifically, this study has 3 practical implications.

Our results show that later patients visit web-based review platforms to seek more information about physicians to decrease the risks of their choices [64]; therefore, it is essential that satisfied reviewers point out their physicians’ shortcomings, in addition to praising them using positive emotions in review texts, to improve their reviews’ helpfulness. Even reviewers who have positive feelings toward their physicians should not remain silent if they are dissatisfied. They should use low scores to indicate deficiencies in certain aspects of their physicians’ services. For dissatisfied reviewers, keeping their sentiments and ratings consistent is essential. Dissatisfied patients should not give high ratings with many complaints because later patients will be confused by reviews with low ratings but positive emotions.

Because patients are concerned about the web-based reviews of doctors, web-based reviews have an indirect impact on physicians. Although higher rankings are important for building reputation and attracting more patients, to help more patients know physicians better, expressing some weaknesses of the physicians’ treatments is useful. For example, when a review ranks a physician with a low score on punctuality, later patients who are in a rush can avoid this physician so that they will not be disappointed because of the long waiting time. Physicians who obtain low ratings in some aspects should not worry too much if patients have positive emotions toward them. Later patients understand that “no one is perfect,” and they can reduce risks by understanding possible negative consequences before treatment.

The current rating mechanism in web-based review platforms is not very efficient for later patients to judge physicians because reviewers and review readers have different perceptions about how satisfactory physicians should be to deserve 5-star ratings. Thus, some ambivalent attitudes in a single review may confuse later patients. Platforms should declare the rules of the ratings or use algorithms to translate patients’ subjective reviews into numerical ratings, rather than asking patients to rate physicians subjectively. Thus, platforms can become more helpful.

### Limitations and Future Directions

First, this study used only 1 data set of 3906 family physicians and general practitioner on RateMDs. Although it is one of the largest web-based physician review platforms and many studies (eg, the study by Gao et al [8]) have used it as the data source [1], physicians treating other diseases and data from other platforms can be used to further test the results. This website is anonymous for reviewers, so we could not explore the impact of review sources. Future research can include reviewers’ traits to better explain the mechanism. This platform does not provide information about whether and how physicians can manage their web-based reputation, and this may also influence the results because physicians may delete unfavorable reviews to enhance their web-based reputation. Future studies can choose other platforms to explore this issue in depth.

Then, we used the number of helpfulness votes as a proxy for review helpfulness and the possibility that the review can be regarded as helpful. Although this proxy is widely used in existing research (eg, the studies by Mudambi and Schuff [18], Cao et al [23], and Filieri et al [30] used the antecedent proxy, whereas the studies by Schlosser [17], Choi and Leon [22], and Pan and Zhang [62] used the later proxy), it is still limited because it reflects patients’ perception of the helpfulness of a review and not the actual helpfulness of the review. Therefore, future studies can explore a better method for measuring how helpful web-based reviews are, such as evaluating whether the reviews have impacts on patients’ final choices of physicians and the degree to which the patient’s uncertainty can be reduced.

Third, we relied on SentiStrength to extract the sentiment score of each review. Although this sentiment analysis tool was widely used in previous research [60] and is one of the best machine learning tools [61] for obtaining the strength of the sentiment in short texts, other tools and methods can also be applied to make a better assessment of sentiments.

Finally, posting web-based reviews is a post hoc behavior that is exhibited after physicians’ treatments, so the behavioral response of the tripartite model of attitudes [47] is ignored in our model. Future research can conduct surveys or experiments to explore whether reviewers’ later choices will be ambivalent to their affective and cognitive attitudes and how this ambivalence will influence later patients’ perceptions.

### Conclusions

In summary, this study focused on ambivalent attitudes in a single web-based review of physicians. Following the tripartite model of attitudes, we conceptualized the numerical rating and text sentiment as the cognitive and affective dimensions of an attitude, respectively, and we confirmed the existence of ambivalence between the 2 dimensions in single reviews. From the risk reduction perspective, we explored how the impact of ambivalent attitudes varies with review valence. We collected 114,378 reviews of 3906 physicians to test our model. The results indicate that for reviews with positive emotional valence, ambivalent attitudes will lead to more helpfulness, but for reviews with negative and neutral emotional valence, ambivalent attitudes will lead to less helpfulness.

## Abbreviations

H1: hypothesis 1
H2: hypothesis 2
OLS: ordinary least squares

## Data Availability

The data used in this study were collected from RateMDs website [65] in May 2019. The sample included patients’ web-based reviews for 3906 family physicians and general practitioner on RateMDs.

## References

[1] Hong YA, Liang C, Radcliff TA, Wigfall LT, Street RL (2019). What do patients say about doctors online? A systematic review of studies on patient online reviews. J Med Internet Res.

[2] Han X, Qu J, Zhang T (2019). Exploring the impact of review valence, disease risk, and trust on patient choice based on online physician reviews. Telematics and Informatics.

[3] Hedges L, Couey C (2020). How patients use online reviews. Software Advice.

[4] Dunivin Z, Zadunayski L, Baskota U, Siek K, Mankoff J (2020). Gender, soft skills, and patient experience in online physician reviews: a large-scale text analysis. J Med Internet Res.

[5] Lu W, Wu H (2019). How online reviews and services affect physician outpatient visits: content analysis of evidence from two online health care communities. JMIR Med Inform.

[6] Grabner-Kräuter Sonja, Waiguny MK (2015). Insights into the impact of online physician reviews on patients' decision making: randomized experiment. J Med Internet Res.

[7] Lin Y, Hong YA, Henson BS, Stevenson RD, Hong S, Lyu T, Liang C (2020). Assessing patient experience and healthcare quality of dental care using patient online reviews in the United States: mixed methods study. J Med Internet Res.

[8] Gao GG, Greenwood BN, Agarwal R, McCullough JS (2015). Vocal minority and silent majority: how do online ratings reflect population perceptions of quality?. MIS Quarterly.

[9] Gao GG, McCullough JS, Agarwal R, Jha AK (2012). A changing landscape of physician quality reporting: analysis of patients' online ratings of their physicians over a 5-year period. J Med Internet Res.

[10] Holliday AM, Kachalia A, Meyer GS, Sequist TD (2017). Physician and patient views on public physician rating websites: a cross-sectional study. J Gen Intern Med.

[11] Emmert M, Sauter L, Jablonski L, Sander U, Taheri-Zadeh F (2017). Do physicians respond to web-based patient ratings? An analysis of physicians' responses to more than one million web-based ratings over a six-year period. J Med Internet Res.

[12] Hu N, Zhang J, Pavlou PA (2009). Overcoming the J-shaped distribution of product reviews. Commun ACM.

[13] Siering M, Muntermann J (2013). Credence goods and online product reviews: an exploration of the product type concept in the social commerce era. Proceedings of the 19th Americas Conference on Information Systems.

[14] Giorgi Rossi P, Ronco G, Mancuso P, Carozzi F, Allia E, Bisanzi S, Gillio-Tos A, De Marco L, Rizzolo R, Gustinucci D, Del Mistro A, Frayle H, Confortini M, Iossa A, Cesarini E, Bulletti S, Passamonti B, Gori S, Toniolo L, Barca A, Bonvicini L, Venturelli F, Benevolo M, NTCC2 Working Group (2022). Performance of HPV E6/E7 mRNA assay as primary screening test: results from the NTCC2 trial. Int J Cancer.

[15] Blenkinsopp A, Wilkie P, Wang M, Routledge PA (2007). Patient reporting of suspected adverse drug reactions: a review of published literature and international experience. Br J Clin Pharmacol.

[16] Paul BR (2020). Mixed reviews: critiques and compliments of physician rating websites. UBC Med J.

[17] Schlosser AE (2011). Can including pros and cons increase the helpfulness and persuasiveness of online reviews? The interactive effects of ratings and arguments. J Consum Psychol.

[18] Mudambi SM, Schuff D (2010). What makes a helpful online review? A study of customer reviews on Amazon.com. MIS Quarterly.

[19] Hong H, Xu D, Wang GA, Fan W (2017). Understanding the determinants of online review helpfulness: a meta-analytic investigation. Decis Support Syst.

[20] Mousavizadeh M, Koohikamali M, Salehan M, Kim DJ (2020). An investigation of peripheral and central cues of online customer review voting and helpfulness through the lens of elaboration likelihood model. Inf Syst Front.

[21] Eslami SP, Ghasemaghaei M, Hassanein K (2018). Which online reviews do consumers find most helpful? A multi-method investigation. Decis Support Syst.

[22] Choi HS, Leon S (2020). An empirical investigation of online review helpfulness: a big data perspective. Decis Support Syst.

[23] Cao Q, Duan W, Gan Q (2011). Exploring determinants of voting for the “helpfulness” of online user reviews: a text mining approach. Decis Support Syst.

[24] Qahri-Saremi H, Montazemi AR (2019). Factors affecting the adoption of an electronic word of mouth message: a meta-analysis. J Manag Inf Syst.

[25] Godes D, Mayzlin D (2004). Using online conversations to study word-of-mouth communication. Market Sci.

[26] Kong D, Yang J, Duan H, Yang S (2020). Helpfulness and economic impact of multidimensional rating systems: perspective of functional and hedonic characteristics. J Consumer Behav.

[27] Malik MS, Hussain A (2017). Helpfulness of product reviews as a function of discrete positive and negative emotions. Comput Human Behav.

[28] Chua AY, Banerjee S (2015). Understanding review helpfulness as a function of reviewer reputation, review rating, and review depth. J Assn Inf Sci Tec.

[29] Quaschning S, Pandelaere M, Vermeir I (2015). When consistency matters: the effect of valence consistency on review helpfulness. J Comput Mediat Comm.

[30] Filieri R, Raguseo E, Vitari C (2020). Extremely negative ratings and online consumer review helpfulness: the moderating role of product quality signals. J Travel Res.

[31] Thompson MM, Zanna MP, Griffin DW, Petty RE, Krosnick JA (1995). Let's not be indifferent about (attitudinal) ambivalence. Attitude Strength: Antecedents and Consequences.

[32] Gao B, Hu N, Bose I (2017). Follow the herd or be myself? An analysis of consistency in behavior of reviewers and helpfulness of their reviews. Decis Support Syst.

[33] Qiu L, Pang J, Lim KH (2012). Effects of conflicting aggregated rating on eWOM review credibility and diagnosticity: the moderating role of review valence. Decis Support Syst.

[34] Cao X, Liu Y, Zhu Z, Hu J, Chen X (2017). Online selection of a physician by patients: empirical study from elaboration likelihood perspective. Comput Human Behav.

[35] Aghakhani N, Oh O, Gregg DG, Karimi J (2020). Online review consistency matters: an elaboration likelihood model perspective. Inf Syst Front.

[36] Chevalier JA, Mayzlin D (2006). The effect of word of mouth on sales: online book reviews. J Market Res.

[37] zhou Y, Yang S, li Y, chen Y, Yao J, Qazi A (2020). Does the review deserve more helpfulness when its title resembles the content? Locating helpful reviews by text mining. Inf Process Manag.

[38] Saifee DH, Zheng Z, Bardhan IR, Lahiri A (2020). Are online reviews of physicians reliable indicators of clinical outcomes? A focus on chronic disease management. Inf Syst Res.

[39] Hao H (2015). The development of online doctor reviews in China: an analysis of the largest online doctor review website in China. J Med Internet Res.

[40] Zhang Z, Liang S, Li H, Zhang Z (2019). Booking now or later: do online peer reviews matter?. Int J Hosp Manag.

[41] Valdivia A, Luzon MV, Herrera F (2017). Sentiment analysis in TripAdvisor. IEEE Intell Syst.

[42] Hu N, Koh NS, Reddy SK (2014). Ratings lead you to the product, reviews help you clinch it? The mediating role of online review sentiments on product sales. Decis Support Syst.

[43] Eagly AH, Chaiken S, Petty RE, Krosnick JA (1995). Attitude strength, attitude structure, and resistance to change. Attitude Strength: Antecedents and Consequences.

[44] Mudambi SM, Schuff D, Zhang Z (2014). Why aren't the stars aligned? An analysis of online review content and star ratings. Proceedings of the 47th Hawaii International Conference on System Sciences.

[45] Nordgren LF, van Harreveld F, van der Pligt J (2006). Ambivalence, discomfort, and motivated information processing. J Exp Soc Psychol.

[46] Sengupta J, Johar GV (2002). Effects of inconsistent attribute information on the predictive value of product attitudes: toward a resolution of opposing perspectives. J Consum Res.

[47] Breckler SJ (1984). Empirical validation of affect, behavior, and cognition as distinct components of attitude. J Pers Soc Psychol.

[48] Kaiser FG, Wilson M (2019). The Campbell paradigm as a behavior-predictive reinterpretation of the classical tripartite model of attitudes. Eur Psychol.

[49] Li X, Wu C, Mai F (2019). The effect of online reviews on product sales: a joint sentiment-topic analysis. Inf Manag.

[50] Pan Z, Lu Y, Gupta S, Hu Q (2020). You change, I change: an empirical investigation of users' supported incremental technological change in mobile social media. Internet Res.

[51] Amirpur M, Fleischmann M, Benlian A, Hess T (2015). Keeping software users on board-increasing continuance intention through incremental feature updates. Proceedings of the 23rd European Conference on Information Systems.

[52] Hong W, Thong JY, Chasalow LC, Dhillon G (2014). User acceptance of agile information systems: a model and empirical test. Journal of Management Information Systems.

[53] Lim KH, Benbasat I (2000). The effect of multimedia on perceived equivocality and perceived usefulness of information systems. MIS Q.

[54] Hennig-Thurau T, Walsh G, Walsh G (2003). Electronic word-of-mouth: motives for and consequences of reading customer articulations on the internet. Int J Electron Commer.

[55] Sheth JN, Venkatesan M (1968). Risk-reduction processes in repetitive consumer behavior. J Market Res.

[56] Goldsmith RE, Horowitz D (2006). Measuring motivations for online opinion seeking. J Interact Advert.

[57] Tsao HY, Chen MY, Lin HC, Ma YC (2019). The asymmetric effect of review valence on numerical rating. Online Inf Rev.

[58] Khan L SentiStrength.

[59] Thelwall M, Buckley KA, Paltoglou G (2012). Sentiment strength detection for the social web. J Am Soc Inf Sci.

[60] Chen L, Baird A, Straub D (2019). Fostering participant health knowledge and attitudes: an econometric study of a chronic disease-focused online health community. J Manag Inf Syst.

[61] Thelwall M, Buckley K, Paltoglou G, Cai D, Kappas A (2010). Sentiment strength detection in short informal text. J Am Soc Inf Sci.

[62] Pan Y, Zhang JQ (2011). Born unequal: a study of the helpfulness of user-generated product reviews. J Retail.

[63] Khurana S, Qiu L, Kumar S (2019). When a doctor knows, it shows: an empirical analysis of doctors’ responses in a QandA forum of an online healthcare portal. Inf Syst Res.

[64] Malik MS, Hussain A (2018). An analysis of review content and reviewer variables that contribute to review helpfulness. Inf Process Manag.

[65] RateMDs.

